# Impact of COVID-19 on workload burden of a complex radiotherapy facility

**DOI:** 10.1007/s11547-021-01338-8

**Published:** 2021-03-01

**Authors:** Giulio Francolini, Isacco Desideri, Giulia Stocchi, Lucia Pia Ciccone, Viola Salvestrini, Pietro Garlatti, Michele Aquilano, Daniela Greto, Pierluigi Bonomo, Icro Meattini, Vieri Scotti, Silvia Scoccianti, Gabriele Simontacchi, Lorenzo Livi

**Affiliations:** 1 CyberKnife Center, Istituto Fiorentino di Cura ed Assistenza, Radiation Oncology Unit, University of Florence, Florence, Italy; 2 Radiation Oncology Unit, Azienda Ospedaliero-Universitaria Careggi, Viale Morgagni 85, 50134 Florence, Italy; 3 Department of Biomedical, Experimental and Clinical Sciences “Mario Serio”, University of Florence, Florence, Italy

## Abstract

**Background and purpose:**

COVID-19 constitutes a worldwide threat, prompting Italian Government to implement specific measures on March 8, 2020, to protect patients and health workers from disease transmission. The impact of preventive measures on daily activity of a radiotherapy facility may hamper the ability to fulfill normal workload burden. Thus, we assessed the number of delivered treatments in a specific observation period after the adoption of preventive measures (since March 11 to April 24, 2020) and compared it with the corresponding period of the year 2019.

**Materials and methods:**

Overall number of delivered fractions was related to actual time of platform daily activity and reported as a ratio between number of delivered fractions and activity hours (Fr/Hrs). Fr/Hrs were calculated and compared for two different periods of time, March 11–April 24, 2019 (Fr/Hrs1), and March 11–April 24, 2020 (Fr/Hrs2).

**Results:**

Fr/Hrs1 and Fr/Hrs2 were 2.66 and 2.54 for year 2019 and 2020, respectively, for a Fr/Hrs^ratio^ of 1.07 (95% CI 1.03–1.12, *p* = 0.0005). Fr/Hrs1 was significantly higher than Fr/Hrs2 for Sli^R^ and Precise^R^, with Fr/Hrs^ratio^ of 1.92 (95% CI 1.66–2.23, *p* < 0.0001) and 1.11 (95% CI 1.03–1.2, *p* = 0.003), respectively. No significant difference was reported for Synergy^R^ and Cyberknife^R^ with Fr/Hrs^ratio^ of 0.99 (95% CI 0.91–1.08, *p* = 0.8) and 0.9 (95% CI 0.77–1.06, *p* = 0.2), respectively. Fr/Hrs1 was significantly lower than Fr/Hrs2 for Tomotherapy^R^, with Fr/Hrs^ratio^ of 0.88 (95% CI 0.8–0.96, *p* = 0.007).

**Conclusion:**

Preventive measures did not influence workload burden performed. Automation in treatment delivery seems to compensate effectively for health workers number reduction.

## Background and purpose

Coronavirus (COVID-19) disease currently constitutes a worldwide threat [1], and pandemic status was declared by World Health Organization on March 11, 2020. Europe faced this issue after the China outbreak, and Italy was one of the most affected countries [2, 3]. Early experiences of emergency management within the framework of a public National healthcare system have already been published [4, 5], but the risk of a widespread propagation of COVID-19 between patients referring to hospitals prompted the Italian Government to implement specific preventive measures on March 8, 2020 [6]. Main actions consisted in temperature check and hand disinfection at departments entrance, use of personal protective equipment (PPE) for all health workers and patients, limitation of caregivers access into hospital and healthcare personnel shortage. Specifically, technical staff (Medical Physics, Radiology Technician, MD) was divided into different working groups to prevent the spread of disease between healthcare personnel reducing inter-operator contacts. Moreover, waiting room for patients was reorganized to reduce and space the seats, limiting the access to patients waiting for their treatment. These measures are particularly important for oncological care, considering that cancer patients are a fragile population, due to the synergistic immunodepressive effects of disease and antineoplastic treatments, with higher risk of COVID-19 and poorer prognosis in this setting [7, 8]. Reduction of population density in the clinical environment (e.g., lower number of health workers and caregivers together) is of utmost importance. However, the impact of preventive measures on daily activity of a radiotherapy facility is a potential issue, hampering the ability to fulfill normal workload burden. This is critical especially considering that oncological treatments (e.g., definitive, adjuvant or palliative radiotherapy) are often non-deferrable. Indeed, the reduction of elective services remains still challenging due to the heterogeneity of cancer treatments [9]. The reduction in terms of output is unacceptable to avoid impact on waiting list of a radiotherapy facility. One of the most intuitive output measures in this context is amount of daily delivered treatments. For this reason, we assessed the number of delivered treatments in a specific observation period after the adoption of preventive measures (since March 11 to April 24, 2020) and compared it with the corresponding period of the year 2019, aiming to assess the impact of COVID-19 prevention on daily routine of our department.

## Materials and methods

Data about delivered treatments and daily time of scheduled activity of all linear accelerators available in Careggi Hospital Radiotherapy Department (Florence, Italy) were collected and reported. Overall equipment consisted in 3 linear accelerators (LINACS): Sli^R^, Precise^R^, Synergy^R^ (Elekta, Stockholm, Sweden), one Tomotherapy^R^ and one Cyberknife system^R^ (Accuray, Sunnyvale, California). Data about Sli^R^, Precise^R^, Synergy^R^ and Tomotherapy^R^ were downloaded from Mosaiq^R^ patients and treatment data management system (Elekta, Stockholm, Sweden). Workload for Cyberknife^R^ system was manually registered from daily schedule. Differences in terms of percentages of treatment fractions delivered on the same platform in the two different periods were tested by comparison of proportions. Overall number of delivered fractions was related to actual time of platform daily activity and reported as a ratio between number of delivered fractions and activity hours (Fr/Hrs). All planned interventions for equipment maintenance and quality assessment occurred in the period analyzed were deducted from overall platform activity time. Fr/Hrs were calculated for two different periods of time, March 11–April 24, 2019 (Fr/Hrs1), and March 11–April 24, 2020 (Fr/Hrs2). Preventive measures for COVID-19 pandemic were adopted on March 8, 2020. Fr/Hrs1 and Fr/Hrs2 were compared through test based method, and their ratio (Fr/Hrs^ratio^) was calculated by Exact Poisson Method. Briefly, a Fr/Hrs^ratio^ > 1 suggested that higher number of fractions for hour of activity were delivered on the same platform in 2019 if compared to 2020. All statistical analyses were performed through MedCalc Statistical Software version 18.9.1 (MedCalc Software bv, Ostend, Belgium).

## Results

Overall, 4267 and 4031 treatment fractions were delivered in a total activity time of 1600 and 1584 h in 2019 and 2020, respectively. Workload burden for each single platform was compared between the two different periods: 12.8 vs 7%, 34.4 vs 33%, 25.6 vs 28%, 8.2 vs 7% and 19 vs 25% of overall amount of treatment fractions were delivered on Sli^R^, Precise^R^, Synergy^R^, Cyberknife^R^ and Tomotherapy^R^ in 2019 vs 2020, respectively. Differences were statistically significant for Sli^R^, Synergy^R^, Cyberknife^R^ and Tomotherapy^R^, with variations between 2019 and 2020 of − 5.8 (95% CI 4.5;7%, *p* < 0.0001), + 2.4 (95%CI 0.49;4.3, *p* = 0.01), − 1.2 (95%CI 0.05;2.34, *p* = 0.03) and + 6% (95%CI 4.2;7.8, *p* < 0.0001), respectively. Conversely, no significant difference was detected for Precise^R^, with − 1.4% (95%CI -0.64;3.4, *p* = 0.17). Fr/Hrs1 and Fr/Hrs2 were 2.66 and 2.54 for year 2019 and 2020, respectively, for a Fr/Hrs^ratio^ of 1.07 (95% CI 1.03–1.12, *p* = 0.0005). In particular Fr/Hrs1 and Fr/Hrs2 were 2.8 vs 1.4, 3.73 vs 3.34, 2.78 vs 2.8, 1.44 vs 1.59 and 2.14 vs 2.44 on Sli^R^, Precise^R^, Synergy^R^, Cyberknife^R^ and Tomotherapy^R^, respectively. Fr/Hrs1 was significantly higher than Fr/Hrs2 for SliR and PreciseR, with Fr/Hrs^ratio^ of 1.92 (95% CI 1.66–2.23, *p* < 0.0001) and 1.11 (95% CI 1.03–1.2, *p* = 0.003), respectively. No significant difference was reported for Synergy^R^ and Cyberknife^R^ with Fr/Hrs^ratio^ of 0.99 (95% CI 0.91–1.08, *p* = 0.8) and 0.9 (95% CI 0.77–1.06, *p* = 0.2), respectively. Conversely, Fr/Hrs1 was significantly lower than Fr/Hrs2 for Tomotherapy^R^, with Fr/Hrs^ratio^ of 0.88 (95% CI 0.8–0.96, *p* = 0.007). Sensitivity analyses were conducted also grouping all platforms equipped only with manual setup corrections (Sli^R^ and Precise^R^), and all platforms were automated setup corrections and/or tumor tracking is available (SynergyR, CyberknifeR and TomotherapyR).

**Fig. 1:**
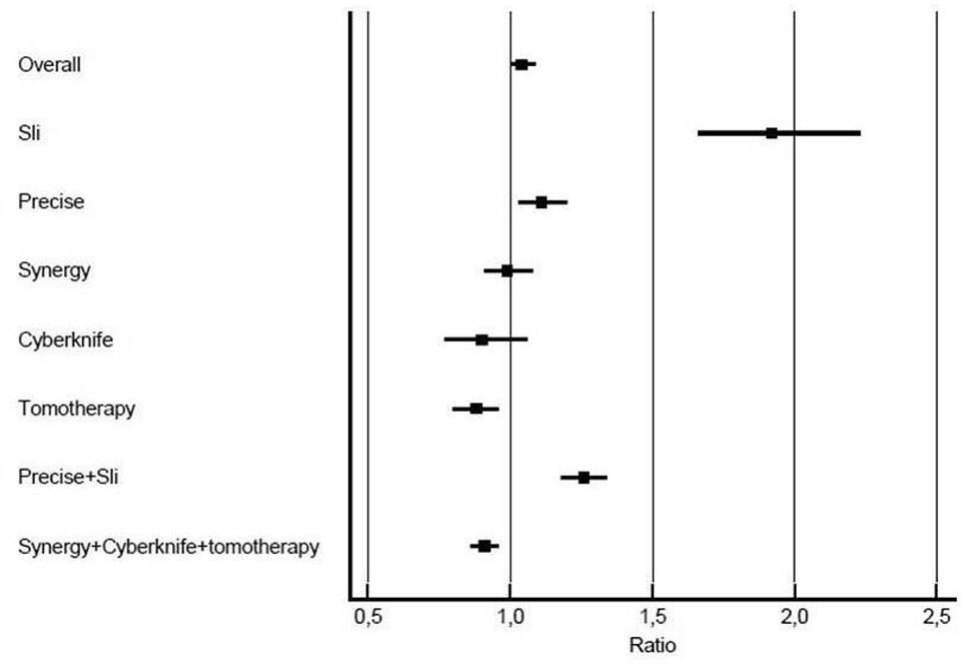
Workload burden performed variation between corresponding periods of years 2019 and 2020, expressed as a ratio between numbers of treatment fractions delivered per hour

Overall, significant difference between 2019 and 2020 was detected for Sli^R^ and Precise^R^, with a 6.4% decrease in terms of delivered treatment fractions (95%CI − 8.6; − 4.33, *p * < 0.0001), as well as for Synergy^R^, Cyberknife^R^ and Tomotherapy^R^, showing a 6.5% increase (95%CI 4.5; 8.6, ***p *** **< 0.0001**). Moreover, results showed that Fr/Hrs1 was significantly higher than Fr/Hrs2 for the first group, with a Fr/Hrs^ratio^ of 1.26 (95% CI 1.18–1.34, ***p***  **< 0.0001**). Conversely Fr/Hrs1 was significantly lower than Fr/Hrs2 for the second group, with a Fr/Hrs^ratio^ of 0.91 (95% CI 0.86–0.96, *p* = 0.002). Main results are summarized in Table 1 and Fig. 1.

**Table 1:** Total workload burden performed of different platforms analyzed

Platform	Total activity time (Hours)^1^	Treatment fractions delivered (n)	Fr/Hrs^2^	Fr/Hrs^ratio3^	*p*
*Overall*					
2019	1600	4267	2.66	1.04 (1.003–1.09)	***P *** **= 0.003**
2020	1584	4031	2.54		
*SLi*					
2019	195	547	2.8	1.92 (1.66–2.23)	***P *** **< 0.0001**
2020	200	291	1.4		
*PRE*					
2019	393	1466	3.73	1.11 (1.03–1.2)	***P *** **= 0.003**
2020	404	1350	3.34		
*Synergy*					
2019	393	1094	2.78	0.99 (0.91–1.08)	*P* = 0.8
2020	404	1132	2.8		
*Cyberknife*					
2019	242	350	1.44	0.9 (0.77–1.06)	*P* = 0.2
2020	175	279	1.59		
*Tomotherapy*					
2019	377	810	2.14	0,88 (0.8–0.96)	***P *** **= 0.007**
2020	401	979	2.44		
*SLi* + *Precise*					
2019	588	2013	3.42	1.26 (1.18–1.34)	***P *** ** < 0.0001**
2020	604	1641	2.71		
*Synergy* + *Cyberknife* + *Tomotherapy*					
2019	1012	2254	2.22	0.91 (0.86–0.96)	***P *** ** = 0.002**
2020	981	2390	2.43		

Bold represent Statistically significant results

^1^Planned maintenance activity is deducted from total activity time

^2^Number of treatment fractions delivered per hours

^3^Ratio between number of treatment fractions delivered per hour in 2019 and 2020

## Discussion

Overall, results seem to suggest a significant change in terms of platform commitment between comparable time periods of years 2019 and 2020. Indeed, significant reduction in terms of treatment fractions delivered with Sli^R^ and Cyberknife^R^ was detected during COVID-19 pandemic, while other platforms (Synergy^R^ and Tomotherapy^R^) registered significant increase in their activity during this period. In our opinion, trend to deliver hypofractionated treatment schedule during the pandemic may have influenced these data. Indeed, palliative treatment schedules consisting in 30 Gy in 10 fractions, usually delivered with a direct posterior field technique through Sli^R^ platform, may have been discouraged in favor of equieffective schedules consisting in 25 Gy in 5 fractions delivered by volumetric modulated arc therapy through Synergy^R^ platform. Thus, a significant amount of treatment fractions may have been shifted from Sli^R^ to Synergy^R^ for this reason. Cyberknife^R^ platform use has been probably affected by the need to avoid any additional procedure (e.g., fiducials placement for tracking purposes) during the pandemic, if compared to year 2019.

Of course, simultaneous integrated boost (SIB) techniques have been encouraged in 2020, aiming to reduce overall treatment duration, contributing to the increase in Tomotherapy^R^ platform use. Impact of hypofractionated schedules and SIB techniques has been important specially to reduce number of treatment fractions for prostate cancer (both in definitive and postoperative setting) and breast cancer (specially in postoperative setting). Data from the present analysis show a significant difference in terms of number of delivered fractions per hour of platform activity, with a 7% decrease during the COVID-19 pandemic if compared to the homologous time period of 2019. This may be related to the lower number of healthcare professionals working at the same time on the different platforms, to the variation in the logistics of facility, to the increased time needed to care for the single patient due to the precautions requested (e.g., PPE dressing and check before and during clinical activity) or to the overall deceleration of tasks due to multiple checks established. However, the overall impact of COVID-19 diffusion preventive measures on the daily workload burden performed was, in the end, sustainable, considering that only 1 treatment fraction was lost for each 14 delivered.

During the first days of the outbreak, many radiotherapy facilities in Italy were forced to significantly reduce their clinical activity [10], and currently it is not known whether special measures undertaken for COVID-19 outbreak will be maintained also after number of cases decrease in our country [6]. However, these data are encouraging, underlining that present organization could be sustained for a radiotherapy department also in the next future, providing the correct social distancing between healthcare workers and patients while keeping a low impact on treatment delay of undeferrable oncological cares. Indeed, experiences in different fields show that patients requiring high-priority interventions may pose challenges in allocation of resources in the current COVID-19 pandemic [11] and that responses to the COVID-19 outbreak have to be carefully optimized [12]. However, impact of preventive measures on workload burden performed and waiting list represent an important issue for cancer management, considering that careful balance should be performed between the oncological risk of delayed cancer intervention versus the risks of COVID-19 to the patient, treating healthcare professionals and the healthcare system [13]. Indeed, the risk of postponing scheduled procedures, focusing only on COVID-19 situation (the so-called distraction effect) may have negative health and social costs [14]. Conversely, this influence could be considered negligible for our department, a complex institution routinely managing both palliative and curative radiotherapy, systemic treatments (chemo- and immunotherapies) and radiometabolites administration. This is a representative scenario of integration between complete oncological care and preventive measures during the COVID-19 pandemic. Moreover, preventive measures seem to have limited impact on satisfaction of patients treated in our department. Indeed, two validated questionnaires (EORTC QLQ-C30, FACIT-TS-G version 1) and 14 specific questions evaluating perception of COVID-19 measures were administered to patients during pandemic period. Results suggest high level of cancer outpatient satisfaction [15] underlining that both clinical activity and patient perception are unchanged despite the current situation. Interestingly, all platforms equipped with systems allowing to correct the setup without accessing to the bunker were not influenced by the actual measures. Probably, the workflow in these cases is not affected because of the lower direct contact of healthcare workers with the patient and the higher automation of treatment delivery. This aspect deserves further consideration, highlighting that referral to centers with the availability of modern radiotherapy platform may help to reduce unnecessary health workers density during clinical activity. Of note, no COVID-19-infected patients were treated in our institution during the outbreak, and workload burden would have probably been negatively influenced in that eventuality. Of course, referral to validated common recommendations will further improve clinical routine and help to reduce unnecessary workload burden. National guidelines as well as practical recommendations regarding radiotherapy during COVID-19 outbreak have been published [16–19]. These data will be interesting especially in the next future, when national healthcare institutions will have to decide whether preventive measures should be discontinued or maintained. Indeed, this setting will require careful evaluation of the balance between the impact of these measures on cancer clinical care and the need to avoid the risk of new infective outbreaks.

## Conclusion

Covid-19 pandemic significantly influenced platforms commitment during year 2020 in our institution. Despite a significant difference in terms of number of fractions delivered per hour of activity, current preventive measures did not influence workload burden performed in our department. Furthermore, automation in treatment delivery seems to compensate effectively for health workers number reduction. These arguments suggest that measures undertaken during COVID-19 outbreak may be sustainable, if needed, without impact on undeferrable oncological care.

## References

[1] Ippolito G, Hui DS, Ntoumi F, Maeurer M, Zumla A (2020). Toning down the 2019-nCoV media hype-and restoring hope. Lancet Respirat Med.

[2] Rosenbaum L (2020). Facing Covid-19 in Italy–Ethics, Logistics, and therapeutics on the epidemic's front line. New England J Med.

[3] Onder G, Rezza G, Brusaferro S (2020). Case-fatality rate and characteristics of patients dying in relation to COVID-19 in Italy. JAMA.

[4] Grasselli G, Pesenti A, Cecconi M (2020). Critical care utilization for the COVID-19 outbreak in lombardy, Italy: early experience and forecast during an emergency response. JAMA.

[5] Spina S, Marrazzo F, Migliari M, Stucchi R, Sforza A, Fumagalli R (2020). The response of Milan's Emergency Medical System to the COVID-19 outbreak in Italy. Lancet (London, England).

[6] Remuzzi A, Remuzzi G (2020). COVID-19 and Italy: what next?. Lancet (London, England).

[7] Liang W, Guan W, Chen R, Wang W, Li J, Xu K, Li C, Ai Q, Lu W, Liang H, Li S, He J (2020). Cancer patients in SARS-CoV-2 infection: a nationwide analysis in China. Lancet Oncol.

[8] Xia Y, Jin R, Zhao J, Li W, Shen H (2020). Risk of COVID-19 for patients with cancer. Lancet Oncol.

[9] Simcock R, Thomas TV, Estes C, Filippi AR, Katz MA, Pereira IJ, Saeed H (2020). COVID-19: Global radiation oncology's targeted response for pandemic preparedness. Clin Translat Radiat Oncol.

[10] Jereczek-Fossa BA, Palazzi MF, Soatti CP, Cazzaniga LF, Ivaldi GB, Pepa M, Amadori M, Antognoni P, Arcangeli S, Buffoli A, Beltramo G, Berlinghieri S, Bignardi M, Bracelli S, Bruschieri L, Castiglioni S, Catalano G, Di Muzio N, Fallai C, Fariselli L, Filippi AR, Gramaglia A, Italia C, Lombardi F, Magrini SM, Nava S, Orlandi E, Pasinetti N, Sbicego EL, Scandolaro L, Scorsetti M, Stiglich F, Tonoli S, Tortini R, Valdagni R, Vavassori V Marvaso G, CODRAL (Board of Directors of Radiation Oncology Departments in Lombardy) network (2020) COVID-19 Outbreak and Cancer Radiotherapy Disruption in Lombardy, Northern Italy. Clinical oncology (Royal College of Radiologists (Great Britain)), 32(7), e160–e161. https://doi.org/10.1016/j.clon.2020.04.007 10.1016/j.clon.2020.04.007 PMC7177150 32354669

[11] Campi R, Amparore D, Capitanio U, Checcucci E, Salonia A, Fiori C, Minervini A, Briganti A, Carini M, Montorsi F, Serni S, Porpiglia F (2020). Assessing the burden of Nondeferrable major uro-oncologic surgery to guide prioritisation strategies during the COVID-19 pandemic: insights from three Italian high-volume referral centres. Eur Urol.

[12] Stedman M, Lunt M, Davies M, Gibson M, Heald A (2020). COVID-19: Generate and apply local modelled transmission and morbidity effects to provide an estimate of the variation in overall relative healthcare resource impact at general practice granularity. Int J Clin Pract.

[13] Wallis C, Novara G, Marandino L, Bex A, Kamat AM, Karnes RJ, Morgan TM, Mottet N, Gillessen S, Bossi A, Roupret M, Powles T, Necchi A, Catto J, Klaassen Z (2020). Risks from deferring treatment for genitourinary cancers: a collaborative review to aid triage and management during the COVID-19 pandemic. Eur Urol.

[14] Cortiula F, Pettke A, Bartoletti M, Puglisi F, Helleday T (2020). Managing COVID-19 in the oncology clinic and avoiding the distraction effect. Ann Oncol Official J Europ Soc Med Oncol.

[15] Desideri I, Francolini G, Ciccone LP, Stocchi G, Salvestrini V, Aquilano M, Greto D, Bonomo P, Meattini I, Scotti V, Scoccianti S, Simontacchi G, Livi, L (2020) Impact of COVID-19 on patient-doctor interaction in a complex radiation therapy facility. Supportive Care Cancer Official J Multinational Assoc Support Care Cancer, pp 1–7. Advance online publication. https://doi.org/10.1007/s00520-020-05793-3 10.1007/s00520-020-05793-3 PMC7531068 33006676

[16] Meattini I, Franco P, Belgioia L, Boldrini L, Botticella A, De Santis MC, Marvaso G, Montesi G, Parisi S, Triggiani L, Lambertini M, Livi L (2020). Radiation therapy during the coronavirus disease 2019 (covid-19) pandemic in Italy: a view of the nation's young oncologists. ESMO open.

[17] You B, Ravaud A, Canivet A, Ganem G, Giraud P, Guimbaud R, Kaluzinski L, Krakowski I, Mayeur D, Grellety T, Lotz JP (2020). The official French guidelines to protect patients with cancer against SARS-CoV-2 infection. Lancet Oncol.

[18] Braunstein LZ, Gillespie EF, Hong L, Xu A, Bakhoum SF, Cuaron J, Mueller B, McCormick B, Cahlon O, Powell S, Khan AJ (2020). Breast radiation therapy under COVID-19 pandemic resource constraints-approaches to defer or shorten treatment from a comprehensive cancer center in the United States. Adv Radiat Oncol.

[19] Thomson DJ, Palma D, Guckenberger M, Balermpas P, Beitler JJ, Blanchard P, Brizel D, Budach W, Caudell J, Corry J, Corvo R, Evans M, Garden AS, Giralt J, Gregoire V, Harari PM, Harrington K, Hitchcock YJ, Johansen J, Kaanders J, Koyfman S, Langendijk JA, Le QT, Lee N, Margalit D, Mierzwa M, Porceddu S, Soong YL, Sun Y, Thariat J, Waldron J, Yom SS (2020). Practice recommendations for risk-adapted head and neck cancer radiotherapy during the COVID-19 pandemic: An ASTRO-ESTRO consensus statement. Radiotherapy Oncol J Europ Soc Therapeutic Radiol Oncol.

